# Serological cross-reactivity among common flaviviruses

**DOI:** 10.3389/fcimb.2022.975398

**Published:** 2022-09-15

**Authors:** Kai Rol Chan, Amni Adilah Ismail, Gaythri Thergarajan, Chandramathi Samudi Raju, Hock Chai Yam, Manikam Rishya, Shamala Devi Sekaran

**Affiliations:** ^1^ Faculty of Medical and Health Sciences, UCSI University, Kuala Lumpur, Malaysia; ^2^ Department of Medical Microbiology, Faculty of Medicine, University of Malaya, Kuala Lumpur, Malaysia; ^3^ Department of Trauma and Emergency Medicine, University Malaya Medical Centre, Kuala Lumpur, Malaysia

**Keywords:** serological cross reactivity, flaviviruses, cross-protection, antibody assays, antibodies

## Abstract

The *Flavivirus* genus is made up of viruses that are either mosquito-borne or tick-borne and other viruses transmitted by unknown vectors. Flaviviruses present a significant threat to global health and infect up to 400 million of people annually. As the climate continues to change throughout the world, these viruses have become prominent infections, with increasing number of infections being detected beyond tropical borders. These include dengue virus (DENV), West Nile virus (WNV), Japanese encephalitis virus (JEV), and Zika virus (ZIKV). Several highly conserved epitopes of flaviviruses had been identified and reported to interact with antibodies, which lead to cross-reactivity results. The major interest of this review paper is mainly focused on the serological cross-reactivity between DENV serotypes, ZIKV, WNV, and JEV. Direct and molecular techniques are required in the diagnosis of *Flavivirus*-associated human disease. In this review, the serological assays such as neutralization tests, enzyme-linked immunosorbent assay, hemagglutination-inhibition test, Western blot test, and immunofluorescence test will be discussed. Serological assays that have been developed are able to detect different immunoglobulin isotypes (IgM, IgG, and IgA); however, it is challenging when interpreting the serological results due to the broad antigenic cross-reactivity of antibodies to these viruses. However, the neutralization tests are still considered as the gold standard to differentiate these flaviviruses.

## Introduction to *Flavivirus*

Characteristically, flaviviruses are RNA viruses that are enveloped and encode a positive single-stranded genome. These 11-kb viruses of the genus *Flavivirus* belong to the family Flaviviridae (Huang et al., 2014). Out of the 53 recognized *Flavivirus* spp., 40 are known to cause human diseases (Gubler et al., 2007). Flaviviruses are transmitted to vertebrates through the bites of infected mosquitoes and ticks, producing disease in animals and human (Gould & Solomon, 2008; Chong et al., 2019). The bite of an infected vector spreads the virus through the blood stream and lymphatics, and replication is seen in many organs. Flaviviruses that are pathogenic for humans include West Nile virus (WNV), yellow fever virus (YFV), Japanese encephalitis virus (JEV), dengue virus (DENV), Zika virus (ZIKV), and tick-borne encephalitis virus (TBEV) (Simmonds et al., 2017). Classification of the virus is complicated due to its extensive geographical distribution and transmission of the vectors.

As of 2015, DENV, JEV, and WNV are the most common *Flavivirus* infections (Daep et al., 2014). These pathogens represent a significant burden of disease, causing about 400 million cases with 100 million symptomatic cases each year with dengue alone (Bhatt et al., 2013). These viruses mainly cause hemorrhagic disease and encephalitis. For instance, the secondary infection with DENV can cause hemorrhagic fever, while infections with neurotropic *Flavivirus* such as JEV, TBEV, and WNV are responsible for viral encephalitis worldwide (Mathengtheng & Burt, 2014; Johnson, 2016). The clinical manifestations of *Flavivirus* infections can range from undifferentiated fever and mild symptoms to more severe conditions that can be fatal. Forty percent of the world’s population is affected by dengue. In the past 50 years, a 30-fold increase in dengue cases have been reported (Kyle & Harris, 2008). It has been reported that DENV infection can lead to dengue shock syndrome (DSS) or dengue hemorrhagic fever (DHF) with 20,000 fatalities every year (Webster et al., 2009). Another *Flavivirus*, JEV, is endemic in the Western Pacific and Southeast Asia, and it is estimated that at least 68,000 cases are reported every year in these regions and the infection may be symptomatic or asymptomatic with a 20%–30% fatality rate (Solomon, 2004).

In tropical and subtropical countries, *Flavivirus* infections represent a global public health problem. This has major economic, social, and individual consequences to those in these regions (Musso & Desprès, 2020). Rapid climate change, improper controlled urbanization, traveling within endemic areas, migration of populations, and extensive deforestation is associated with flaviviruses adapting to new habitats and host species. These are the main factors that contribute to the rising trend of *Flavivirus* infections and transmission of viruses into previously non-endemic areas (Pandit et al., 2018; Argondizzo et al., 2020). These are exemplified by ZIKV emergence in South America in 2015 and Europe in 2019 (Khawar et al., 2017; Giron et al., 2019), outbreaks of WNV in Europe and North America from the 2000s (Tsai et al., 1998; Nash et al., 2001), and YFV outbreaks in Brazil and Africa (Kraemer et al., 2017; Hamer et al., 2018).

### *Flavivirus* genomic organization

**Figure 1:**
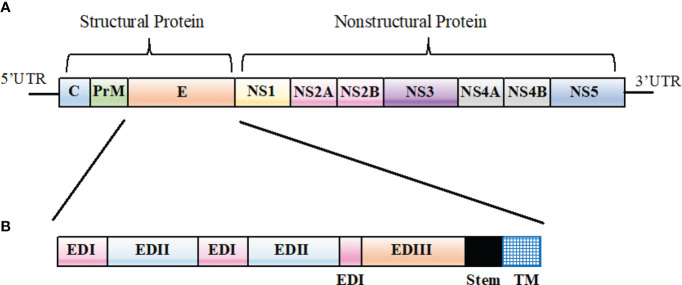
*Flavivirus* genomic organization. **(A)** A schematic representation of *Flavivirus* genomic organization. **(B)** Primary structure of *Flavivirus* E protein ectodomain showing EDI (pink), EDII (light blue), and EDIII (orange). The stem region and the transmembrane (TM) domain represented in black and blue, respectively.

Among flaviviruses, genome organization is similar. Basically, the genome encodes three structural proteins (capsid [C], pre-membrane [prM], and envelope [E]) and seven non-structural (NS) proteins (NS1, NS2A, NS2B, NS3, NS4A, NS4B, and NS5) as shown in **Figure 1**. The structural and non-structural proteins are essential in the formation of virus particles, viral replication, viral polyprotein processing, and cell receptor binding and entry (Mazeaud et al., 2018; Chong et al., 2019).

#### Cross-reactive epitopes of *Flavivirus* E protein

Flaviviruses enter target cells through the interaction of viral E protein with the host surface receptors. Thus, the main antigenic target for antibody responses to DENV is the E protein, which is the most exposed protein on the virus and considered as the main antigenic target for antibody responses in patients (Pierson et al., 2008; Beltramello et al., 2010; Xu et al., 2012). The E protein is made up of three domains (EDI, EDII, and EDIII), and it is EDIII that interacts with attachment factors and receptors (Pierson & Kielian, 2013). Potent neutralizing antibodies are induced by EDIII due to the presence of important antigenic epitopes with strong antigenicity. EDIII is also a common antigen used in serological diagnosis (Chávez et al., 2010; Matsui et al., 2010). On the other hand, anti-EDI and anti-EDII antibodies are less potent but show a broader cross-reactivity among different strains of flaviviruses than those against EDIII (Sun et al., 2017).

Venkatachalam and Subramaniyan (2014) performed multiple sequence alignment of E protein of the four DENV serotypes to study the conservation and homology of amino acids. Their results indicated a close relationship between DENV1 and DENV3, which showed the highest percentage of homology of up to 78.4%. DENV1 with DENV2 and DENV2 with DENV3 showed 66.1% and 66.3% amino acid identity, respectively, while DENV1 with DENV4, DENV2 with DENV4, and DENV3 with DENV4 showed 63.3%, 62.8%, and 63.4% homology, respectively, indicating that DENV4 showed a distant relationship with the three serotypes. Other similar studies using multiple sequence alignment of E protein among flaviviruses showed that the similarity between YFV and TBEV are highly related but distant from the four DENV, WNV, JEV, and ZIKV (Chakraborty, 2016). Other than that, it has been reported that the E protein of ZIKV and DENV2 share ~54% amino acid sequence identity (Priyamvada et al., 2016; Stettler et al., 2016). ZIKV also shares 57% amino acid sequence identity with WNV, 56.1% with JEV, 55.6% with DENV, and 46% with YFV (Chang et al., 2017). A phylogenetic study by Kuno et al. (1998) indicated that non-vector and vector-borne virus clusters emerged from a putative ancestor, and it is from the vector-borne cluster that tick- and mosquito-borne virus clusters emerged. Their observations suggested that the viruses of this genus evolved from non-vector group to tick-borne and then to mosquito-borne group.

**Figure 2:**
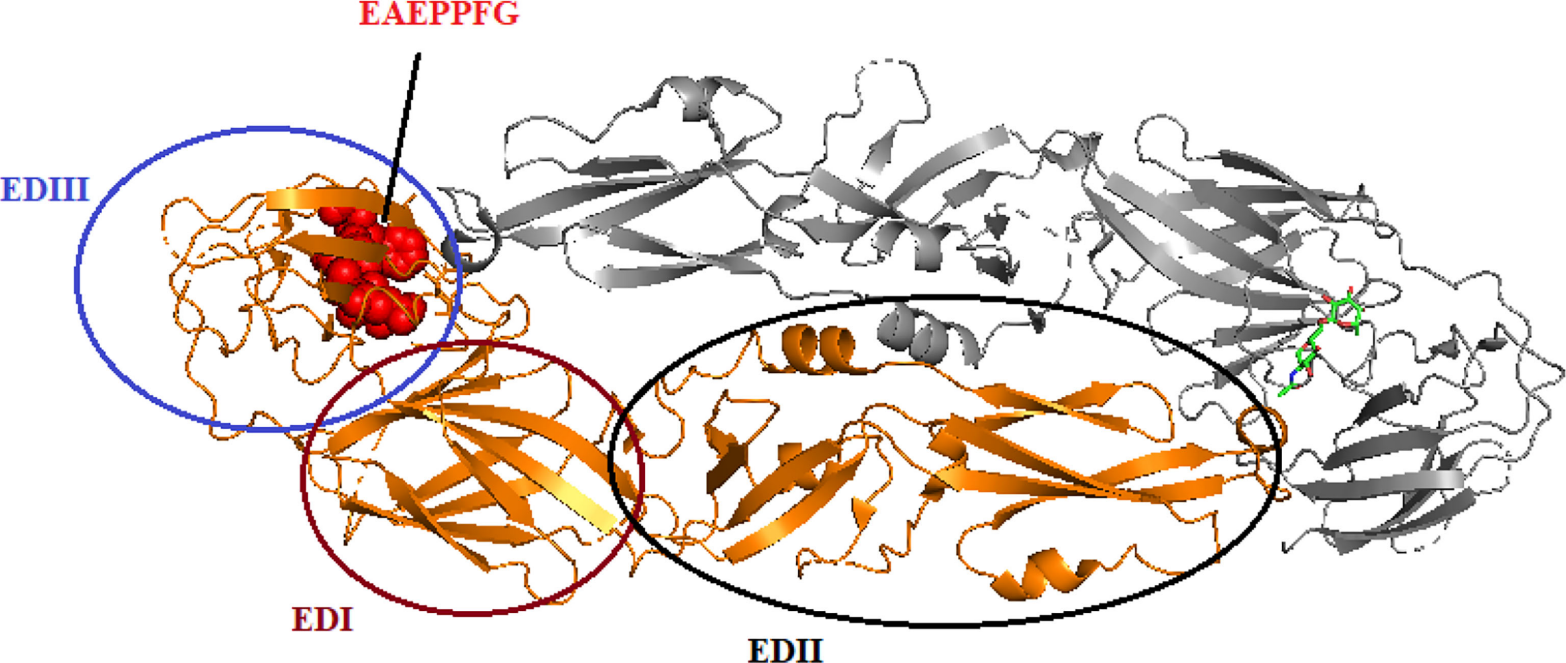
The protein-dimer structure of DENV1 E protein and EAEPPFG epitope located at domain III. The brown, black, and blue circle represent the domain I, domain II, and domain III, respectively, in one E protein monomer. Another monomer is colored gray. The red spheres located in EDIII represent the EAEPPEG epitope.

It had been reported by Li et al. (2016) that the monoclonal antibody (mAb) 3B6 binds to the EXE/DPPFG epitope in E protein domain III, which is highly conserved (approximately 85%) in DENV, WNV, ZIKV, JEV, Murray Valley encephalitis virus (MVEV), and Saint Louis encephalitis (SLEV), indicating that the function of the epitope is similar in these viruses. The cross-reactivity of DENV-, YFV-, ZIKV-, and WNV-positive sera suggested that the EXE/DPPFG epitope is an immunodominant epitope among flaviviruses. The sequences of the E protein of DENV serotypes, WNV, JEV, MVEV, and SLEV were obtained from National Center for Biotechnology Information (NCBI), and the multiple sequences alignment are shown in **Table 1**. The dashes indicate identical amino acids, while the EXE/DPPFG epitope region is indicated by gray shading. **Figure 2** shows the molecular structure of DENV1 E protein and the EXE/DPPFG epitope located at EDIII.

**Table 1:** Sequence alignment of highly conserved flaviviruses E protein EXE/DPPFG epitope.

Epitope	Accession No.	Sequence
DENV1	NP_722460.2	361 KEKPVNIEAE―PPFGESYIVVGAGEKALKLS 390
DENV2	NP_739583.2	360 EKDSPVNIEAE―PPFGDSYIIIGVEPGQLKLN 390
DENV3	YP_001531168.2	358 KKEEPVNIEAE―PPFGESNIVIGIGDNALKIN 388
DENV4	NP_740317.1	360 NTNSVTNIELE―PPFGDSYIVIGVGNSALTLH 390
WNV	YP_001527880.1	366 TANAKVLIELE―PPFGDSYIVVGRGEQQINH 395
WNV	AEB66112.1	366 TANAKVLIELE―PPFGDSYIVVGRGEQQINH 395
JEV	NP_059434.1	366 ANSKVLVEME―PPFGDSYIVVGRGDKQINH 394
JEV	AAA67173.1	366 ANSKVLVEME―PPFGDSYIVVGRGDKQINH 394
YFV	NP_740305.1	358 TNDDEVLIEVN―PPFGDSYIIVGRGDSRLTYQ 388
YFV	QHB50138.1	358 TNDDEVLIEVN―PPFGDSYIIVGTGDSRLTYQ 388
MVEV	NP_722531.1	367 ANAKVLVEIE―PPFGDSYIVVGRGDKQINHH 396
SLEV	YP_009329949.1	367 ANNKVMIEVE―PPFGDSYIVVGRGTTQINYH 396

**Figure 3:**
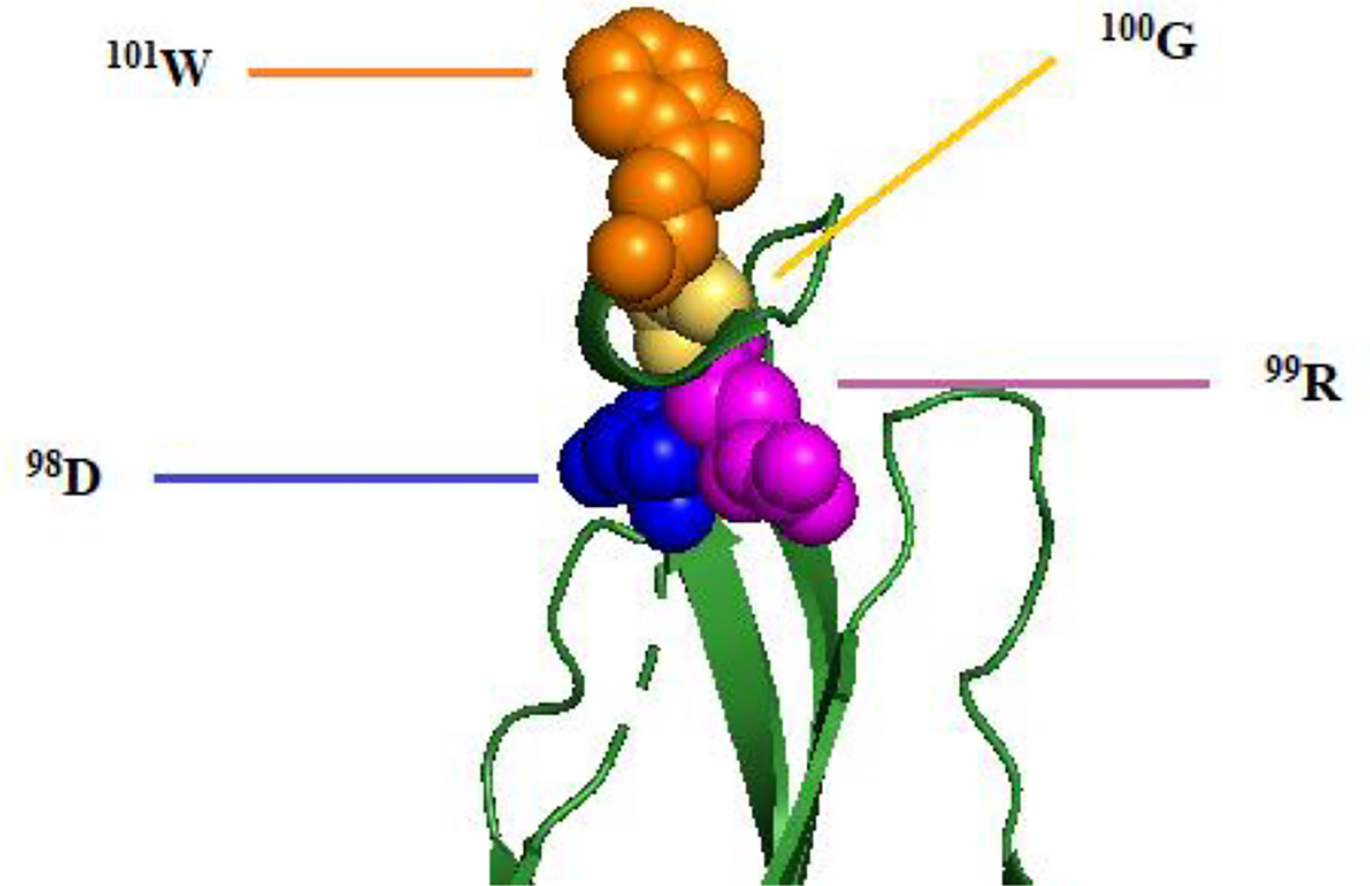
Structure of 2A10G6 epitope of DENV1 E protein. The ^98^DRXW^101^ motif is labeled and colored as blue, purple, yellow, and orange, respectively, according to the residue sequence.

Other than that, Deng et al. (2011) reported that the mAb 2A10G6, which potently neutralizes DENV serotypes, TBEV, JEV, WNV, and YFV, showed a board cross-reactivity to the ^98^DRXW^101^ motif, which is the highly conserved *Flavivirus* fusion loop peptide. The comparison of 2A10G6 epitope of these flaviviruses is shown in **Table 2**, and the epitope region is indicated by gray shading. **Figure 3** shows the structure of 2A10G6 epitope in DENV1, which is located at EDII.

**Table 2:** Comparison of flaviviruses E protein sequence on the 2A10G6 epitope.

Epitope	Accession No.	Sequence
DENV1	NP_722460.2	90 FVCRRTFVDRGWGNGCGLFGKGSLITC 116
DENV2	NP_739583.2	90 FVCKHSMVDRGWGNGCGLFGKGGIVT 115
DENV3	YP_001531168.2	90 YVCKHTYVDRGWGNGCGLFGKGSLVT 115
DENV4	NP_740317.1	90 YICRRDVVDRGWGNGCGLFGKGGVVT 115
WNV	YP_001527880.1	90 FVCRQGVVDRGWGNGCGLFGKGSIDT 115
WNV	AEB66112.1	90 FVCRQGVVDRGWGNGCGLFGKGSIDT 115
JEV	NP_059434.1	90 YVCKQGFTDRGWGNGCGLFGKGSIDT 115
JEV	AAA67173.1	90 YVCKQGFTDRGWGNGCGFFGKGSDT 115
YFV	NP_740305.1	90 NACKRTYSDRGWGNGCGLFGKGSIVA 115
YFV	QHB50138.1	90 NACKRTYSDRGWGNGCGLFGKGSIVA 115
MVEV	NP_722531.1	90 YLCKRGVTDRGWGNGCGLFGKGSIDT 115
SLEV	YP_009329949.1	90 FVCKRDVVDRGWGNGCGLFGKGSIDT 115

There are several cross-reactivity epitopes on the surface of flaviviruses E protein that determine antibody binding and neutralization properties. **Table 3** below summarizes some epitopes and residues that were located at different domains of E protein that show cross-reactivity among flaviviruses.

**Table 3:** *Flavivirus* cross-reactive epitopes and residues located in E protein.

Epitopes	Location	References
A1	EDII, contains at least two independent and overlapping group-reactive epitopes, incorporating two or three of highly conserved fusion peptide residues Gly_104_, Gly_106_, and Leu_107_.	(Crill and Chang, 2004)
A5	EDII, centers on conserved Trp_231_ and structurally related with neighbors Glu_126_ and Thr_226_.	
E111	EDIII AB loop, EDIII residues 314–319.	(Sukupolvi-Petty et al., 2007)
E114	EDIII	
4E11	EDIII, residue 307, 308, 309, 310, 311, 312, 387, 389, and 391.	(Lisova et al., 2007)
	EDIII, residue 306, 308, 381, 387, and 389.	(Matsui et al., 2009)
4G2-1	EDII, residue 101, 104, 106 and 107.	(Chiou et al., 2012)
4G2-2	EDIII, residue 312, 315, 331, 332, and 389.	
6B3B-3, 6B6C-1	EDIII, residue 312, 315, 329, 331, 332, and 332.	
23-1, 23-2	EDII, residue 101 and 107	
5-2	EDI, residue 138.	

### Diagnosis of *Flavivirus*-associated human disease

In the diagnosis of *Flavivirus*-associated human disease, laboratory testing is required. The tests used either directly detected the infecting agent or through the detection of antibodies targeting the infecting virus. The advantages and limitations of several laboratory techniques used for the diagnosis of *Flavivirus* are shown in **Table 4**.

**Table 4:** Methods for the diagnosis of human *Flavivirus* infections.

Methods	Advantages	Limitations	References
Virus isolation	Direct pathogen detection Most specific and conclusive diagnosis	Time consuming Laborious Requirement of acute sample Biosafety Laboratory considerations of level 2–4	(Goncalves et al., 2017) (Musso and Desprès, 2020) (Samuel Sulca Herencia, 2019)
RT-PCR	Detection of viral nuclei acids High sensitivity and specificity Rapidity	Require careful handling to prevent cross-contamination Require specialized instrumentation Expensive	
Viral antigen capture	Detection of acute of DENV based on the capture of soluble NS1 Easy to perform	Only available for DENV Less accurate than viral isolation Requirement of acute sample	
Serology	Detection through the capture of IgG/IgM or virus neutralization assays Qualitative and quantitative serological diagnosis tests	Limited specificity and sensitivity False interpretation of DENV diagnostic might occur during the secondary DENV infection	

Cross-reactivity among flaviviruses was first shown with complement fixation tests (Casals, 1957) followed by the hemagglutination-inhibition assay (Casals & Brown, 1954). Virus-neutralizing tests have strengthened this concept of cross-reactivity and enabled segregation of flaviviruses into the different arthropod-borne viruses and those with unknown arthropod vectors (Calisher et al., 1989). The antigenic similarities between flaviviruses indicate one aspect of the similarities observed among flaviviruses. Both species-specific and *Flavivirus* cross-reactive antibodies are produced when infection with one *Flavivirus* occurs. The majority of flaviviruses that are relevant to human disease were organized into eight serocomplexes that cause cross-reactive immune responses (Rathore & St. John, 2020) consistent for multiple mammalian species (Saron et al., 2018). This cross-reactivity is said to be not durable and hence not retained (Collins et al., 2017). Multiple exposures to various flaviviruses make it extremely difficult to determine the latest infection (Mansfield et al., 2011). This high degree of cross-reactivity further emphasizes the need for virological confirmation.

The detection of the viral genome using PCR-based techniques are highly specific but require clinical laboratories with advanced technology. Despite the broad antigenic cross-reactivity of anti-*Flavivirus* antibodies, serological assays are still widely used to detect different immunoglobulin isotypes (IgM, IgG, and IgA) (Allwinn et al., 2002; Mansfield et al., 2011; Papa et al., 2011). However, the interpretation of these serological results is challenging. The likelihood of experiencing multiple *Flavivirus* infections has resulted in a need to understand the effects of pre-existing immunity during a lifetime and its impact on subsequent exposures. Animal models have been used, and here, however, prior exposures were shown to be protective (Tesh et al., 2002) within the same serocomplex (Williams et al., 2001). However, for DENV, protection is seen only with the homologous serotype (Sabin, 1952). Recent studies (Saron et al., 2018) using JEV showed cross-protection against dengue viruses with increased neutralizing antibodies. With ZIKV, both cross-protection and immune-mediated pathology were noted (Rathore et al., 2019). Therefore, the major interest of this review paper is in characterizing the serological cross-reactivity of DENV serotypes, ZIKV, WNV, and JEV.

## *Flavivirus* serological assays

Currently, there are several serological assays that are able to determine antibody levels to flaviviruses such as the Western blotting assay, dot-blot assay, neutralization tests, hemagglutination-inhibition tests, IgM/IgG antibody-capture ELISAs, immunofluorescent tests, microsphere immunoassays, high-throughput and rapid microneutralization assays, lateral flow tests, biosensors and microfluidic systems, and autologous red blood cell agglutination tests (Hobson-Peters, 2012; Chong et al., 2019).

### Neutralization tests

Neutralizing anti-*Flavivirus* antibodies usually target highly accessible epitopes, and the detection of neutralizing anti-*Flavivirus* antibodies is correlated with the presence of the specific IgG in blood specimens (Roehrig et al., 2008). Neutralization tests are the most reliable serological assay and are capable of providing high specificity among flaviviruses (Russell & Nisalak, 1967). The plaque reduction neutralization test (PRNT), micro-neutralization test (MNT), and virus neutralization test (VNT) are considered as the gold standard in quantifying and detecting the levels of neutralizing antibodies against flaviviruses (Thomas et al., 2009; Guy et al., 2010; Plotkin, 2010). MNT and VNT are the alternative methods of PRNT, using 96-well microplates in the combination with enzyme-linked immunoassays, which are cost effective and widely automated (Li et al., 2017; Nurtop et al., 2018).

Calisher et al. (1989) stated that the PRNT test is the most specific serological test to differentiate the infections caused by flaviviruses in convalescent sera samples. The PRNT test can be carried out in a test tube or microtiter plate. A serial dilution of the serum sample is mixed with a standardized amount of virus. The mixture is then tested for unbound virus by testing for viral infectivity on virus-susceptible cells. Each virus that initiates a productive infection can then be measured by first restricting the spread of progeny virus by overlaying cells with a semi-solid media. This results in the formation of a localized area of infection (a plaque) that can be detected through a variety of ways. The number of plaques is calculated and compared to the initial concentration of virus to determine the percent reduction in total virus infectivity (Zannoli et al., 2018). The PRNT titer is the titer that results in a reduction of 50%–90% plaques.

However, since PRNT is a labor-intensive, time-consuming, and largely manual assay, it is challenging during the diagnosis and is expensive especially when conducting large clinical trials that might involve the testing of hundreds to thousands of samples under good clinical laboratory practice conditions (WHO, 2007; Maeda & Maeda, 2013). In addition, neutralization tests require live virus, as the assay antigen, to detect neutralizing antibodies. This has high variability between assays and between laboratories due to the differences in cell lines used, the maturation state of virus, the strain, and other variations (WHO, 2007; Thomas et al., 2009). Moreover, the laboratories that do not have access to live infectious viruses or do not meet appropriate biosafety standards are unable to perform neutralization tests.

For DENV, PRNT is the most widely accepted method in measuring the virus-neutralizing antibodies (Roehrig et al., 2008). Thomas et al. (2009) conducted a series of assays with different cell lines, virus preparation, and the presence or absence of complement to determine the variability in anti-dengue virus PRNT assays and found that modification of these conditions caused significant effects on PRNT titers, which can be impacted not only by changing a single condition but also by interaction between two or more conditions. Other than that, Rainwater-Lovett et al. (2012) had carried out a systematic review of human PRNT titers to DENV and concluded some additional factors that may influence the PRNT titers. First, the geographical areas of study population affect not only the strains that a person is exposed to but also strains against which their serum was tested. Second, based on the laboratory methods and inclusion criteria on each study, there is likely a link between the testing strains and primary or secondary DENV exposure. Lastly, the quality, duration, and magnitude of antibodies response induced by different infecting strains may differ, highlighting the differences between strains that call for further exploration.

The PRNT is useful for epidemiological or diagnostic purposes as a reduction in plaque counts of ≥80% can ensure prevention of cross-reactivity among flaviviruses in dengue endemic areas. However, the PRNT_50_ titer is the preferred value during the evaluation of vaccines for human use because it affords acceptable sensitivity and specificity (WHO, 2007). Timiryasova et al. (2013) had evaluated several parameters to produce reliable test performance to support the vaccine development program and validated assay reagents to ensure that they are suitable for intended use and consistently performing within the established limits. Their study showed that the dengue PRNT_50_ for each of the serotypes of DENV is precise, accurate, and specific, and it is suitable for detecting and measuring specific neutralizing antibodies to each DENV serotypes in human serum samples with a lower limit of quantitation of 10. However, for dengue, PRNT remains as the gold standard neutralization assay and is recommended by WHO against which any new assay will need to be validated.

Among the flaviviruses, the exposed surfaces show the highest degree of variation. The most potent neutralizing epitopes are type specific, while those that promote antibody-dependent enhancement of infection (ADE) belong to the cross-reactive group (Rey et al., 2017). Of all the structural domains of the E protein, the highly variable EDIII has the most potent neutralizing activity (Stettler et al., 2016; Zhao et al., 2016). However, these areas make up a small component of the human response against WVN and DENV (Throsby et al., 2006; Beltramello et al., 2010) and do not contribute significantly to the neutralization activity present in serum (Wahala et al., 2009). Patients infected with ZIKV who have high anti-EDIII titers have also EDIII-specific neutralizing activity, indicating the major role of these antibodies in ZIKV immunity. Some cross-reactivity with DENV from memory cells was noted in previously DENV-infected patients (Robbiani et al., 2017; Yu et al., 2017). Other than that, there are also quaternary epitopes restricted to the E–E dimer interface, and antibodies to these epitopes have also been described in WNV-, JEV-, and ZIKV-infected patients (Kaufmann et al., 2010; Hasan et al., 2017; Qiu et al., 2018). Other regions implicated include the fusion loop and the prM, but these areas are highly conserved between DENV and ZIKV (Dejnirattisai et al., 2014; Rouvinski et al., 2015), and antibody-escape mutations do not readily develop (Abbink et al., 2018). Recently, cross-reactive antibody responses were shown to promote ADE during the recent ZIKV outbreak in areas with high DENV exposure. This may have been due to the existing anti-DENV immunity and hence may also occur against a wide variety of other flaviviruses (Halstead et al., 1980; Fagbami et al., 1987). Evidence for this response was shown by passive transfer of cross-reactive antibodies isolated from ZIKV- and DENV-infected patients in AG129 mice (Stettler et al., 2016). Other studies conducted were in Stat2^−/−^ mice after intraperitoneal administration of DENV or WNV immune sera (Bardina et al., 2017). In other human studies, it was observed that following JEV vaccination, the cross-reactive antibodies induced was found to be associated with an increased risk of symptomatic dengue illness (Anderson et al., 2011; Saito et al., 2016). However, evidence obtained from many other studies showed otherwise with no effect in ZIKV-immune macaques (George et al., 2017), whether exposed to DENV or YFV (McCracken et al., 2017). Recently, a study of pregnant women infected with ZIKV indicated that the presence of DENV antibodies did not have any significant association with congenital Zika syndrome (Halai et al., 2017).

Cross-reactivity of anti-Powassan virus (POWV) antibodies against other tick-borne flaviviruses (TBFVs) and mosquito-borne flaviviruses (MBFVs), which include TBEV, Gadgets Gully virus (GGYV), Langat virus (LGTV), SLEV, ZIKV, WNV, YFV, and Usutu virus (USUV), was shown in a study conducted by VanBlargan et al. (2021). Their results revealed that the anti-POWV antibodies were cross-reactive with other flaviviruses and cross-neutralized other TBFVs. Moreover, the studies stated that the cross-reactivity and cross-neutralization of some POWN EDIII-specific mAbs occurred because of the recognition of the lateral ridge/C–C’ loop epitope and thus were protective against TBEV and LGTV. This was similarly shown with ZIKV mAbs, which targets the epitopes on the EDIII-LR and C–C’ loop on ZIKV, which cross-reacted with DENV (Sapparapu et al., 2016; Robbiani et al., 2017; Zhao et al., 2019). Therefore, the EDIII-LR/C–C’ loop epitope represents an antigenic site with multiple TBFV and MBFV areas that can be targeted.

### Enzyme-linked immunosorbent assay

The ELISA is a technique that enables many laboratories to test numerous samples simultaneously. It is used in quantifying and detecting a specific protein in a complex mixture. ELISAs are performed in a 96- or 384-well plate coated with antibodies and proteins passively bound in the well. There are several formats used for ELISAs, such as direct, indirect, or sandwich methods for capture and detection. The most important step is the attachment of the antigen to the assay microtiter plate, and this is done by either direct adsorption to the assay plate or indirectly *via* a capture antibody that has been attached to the plate. The antigen is then detected either directly (labeled primary antibody) or indirectly (such as labeled secondary antibody). The most widely used ELISA assay format is the sandwich ELISA assay due to its specificity and sensitivity (ThermoFisher, 2010). However, ELISA is labor-intensive to carry out, and the cost for the preparation of the antibody is high because it is a sophisticated technique that requires cell culture media to obtain a specific antibody. It is important to note that errors occur when there is insufficient blocking of the surface of microtiter plate immobilized with antigen and this can lead to high false-negative or false-positive results (Sakamoto et al., 2017).

IgM antibody capture ELISA (MAC-ELISA) can be used in the diagnosis of DENV infections by detecting specific IgM antibodies in serum samples. In the early acute phase of the disease, there is a negative window period of detection, as the antibody response has not been mounted. IgM can only be detected on days 3–5 after the onset of the disease (Shu & Huang, 2004). Other than that, during the acute phase of DENV infection or primary *Flavivirus* infection, IgG is undetectable. However, IgG can be detected after 3 days of the onset of the disease during secondary infection (Kit Lam et al., 2000). Therefore, the IgM : IgG ratio can be used to differentiate primary from secondary infections of the disease. Fox et al. (2011) had stated that the IgM : IgG ratio, which is equal or above 1.8, is defined as primary DENV infection, while the ratio below 1.8 is defined as secondary DENV infection. It needs to be stated that it is not advisable to use IgM alone, as it lingers in the body for more 90 days. Diagnosis based on IgM alone is usually not confirmatory.

The DENV NS1 detection ELISA was first developed in 2000. NS1 is found both as membrane bound and in soluble forms and is highly conserved (Young et al., 2000). Importantly, NS1 protein is detectable earlier during the acute phase of both primary and secondary DENV infections (Libraty et al., 2002; Duyen et al., 2011). Alcon et al. (2002) and Shu et al. (2002) reported that the NS1 protein was detectable from day 1 to 8 after the onset of illness. Therefore, NS1 detection ELISA allows for the early serological diagnosis of virus infection. The commercially available NS1 detection ELISA kits such as the Pan-E Dengue Early ELISA from Panbio (Kit Pan-E) and Platelia Dengue NS1 Ag from Bio-Rad (Kit Platelia) were evaluated by several studies (Chuansumrit et al., 2008; Guzman et al., 2010; Lima et al., 2010; Pal et al., 2014; Hunsperger et al., 2014). Guzman et al. (2010); Lima et al. (2010) and Pal et al. (2014) had reported that the overall diagnostic sensitivity and specificity of Platelia was higher than that of Pan-E, and the sensitivity was better before day 4 after the onset of disease. However, the sensitivity for serotype DENV-2 and DENV-4 were lower in both studies. The inclusion of samples from YFV and JEV infections suggested that there was no cross-reaction among flaviviruses, while the lower specificity of kit Pan-E was due to the false-positive results in patients with JEV, YFV, and acute influenza infections (Guzman et al., 2010). Several studies had reported that the sensitivity of Platelia was lower in secondary infections in comparison to primary infections (Osorio et al., 2010; Duong et al., 2011; Pal et al., 2014) due to the production of anti-NS1 antibodies, which was detected more frequently during the DENV secondary infection (Koraka et al., 2003), and the formation of immune complexes impeded the detection of free NS1 protein (Young et al., 2000; Libraty et al., 2002). Duong et al. (2011) reported that overall sensitivity increased when the NS1 antigen assay is coupled with MAC-ELISA, which allows definitive (NS1) or presumptive (IgM) diagnosis during the acute and convalescent phase of the disease and can be used as a “point of care” diagnosis.

DENV and ZIKV share genetic and antigenic determinants (Breitbach et al., 2019), which cause difficulties in serological assays. Denis et al. (2019) observed that ZIKV EDIII, which is specifically recognized by anti-ZIKV IgG, is able to distinguish ZIKV from DENV in the late phase with high sensitivity, and thus, they developed and carried out a study on ZEDIII recognition by IgG with more than 5,000 serum samples. They found out that the ZEDIII protein sequence shares 46.3% and 47.2% identity and 64.8% homology with the protein sequences of DENV-2 EDIII and DENV-4 EDIII. Then, they tested the ability of ZEDIII-immunized mouse sera to detect the recombinant EDIII to further validate the high specificity of humoral immune response towards EDIII, and their results showed that the sera of ZEDIII-positive mice did not recognize DEN-4EDIII. They found out that the purified human DEN-2EDIII-IgG or DEN-4EDIII-IgG did not cross-react with ZEDIII, and the epitopes that are recognized by ZEDIII-IgG are different. Moreover, Sapparapu et al. (2016) had reported that the ZIKV EDIII antibodies tested by ELISA did not bind to WNV-EDIII or DEN-2EDIII. Denis et al. (2019) compared their ZEDIII-based ELISA with NS1-based ELISA and found out that the sensitivity and specificity of NS1-based ELISA was higher. It has been suggested that the ZEDIII could be used a safe model for the development of vaccines.

### Hemagglutination-inhibition test

Hemagglutination inhibition test (HI) has been used in the diagnosis of DENV for many years since 1950 when Sabin (1950) discovered that arboviruses are able to agglutinate certain types of red blood cells. Traditionally, HI test is used to differentiate between the primary and secondary DENV infections (WHO, 1997). Some viruses cause hemagglutination, and this property is used in the HI test. The absence of hemagglutination indicates the presence of antibodies, as these would have bound to the virus and prevented hemagglutination. HI test can be used to detect the antibodies in the case of a known hemagglutinating virus. On the other hand, for the case of unknown viruses, a panel of known antibodies can be used to identify the virus. The HI test has the advantage of being easy to perform. However, the HI test might not detect the antibodies that are not cross-reactive to the viral subtype being tested; thus, all possible subtypes of a virus should be included during the HI test to effectively detect the antibodies, which is not always feasible (Bourgeois & Oaks, 2014).

Anantapreecha et al. (2007) had conducted a study in Thailand to analyze the level of DENV cross-reactivity of HI antibodies in primary DENV infections. A total number of 101 confirmed cases of DENV 1–4 were selected, and the plasma samples were obtained twice, once during the acute phase and once during the convalescent phase. Their results showed that the HI antibodies were cross-reactive among four DENV serotypes during both acute and convalescent phase in the primary infection, which indicated that the HI test is not the best method to differentiate different serotypes of DENV due to their cross-reactivity. Other than that, several studies reported that the HI test showed a low performance in distinguishing secondary DENV infection (Graham et al., 1999; Atchareeya et al., 2006; Lukman et al., 2016). It has been reported that the HI test is unable to differentiate among the flaviviruses DENV, JEV, and WNV (Nisalak, 2015). Several studies had compared the HI test with ELISA assays and stated that the ELISA assay was more reliable than the HI test for discrimination of primary and secondary DENV infection (Vaughn et al., 1999; Matheus et al., 2005; Atchareeya et al., 2006; De Souza et al., 2007). It is important to note that HI can still be used, but the criteria for interpretation needs to be carefully considered. Any titer below <80 should be classified as primary infection, and those between 60 and 640 of both the serum pairs must be used before a classification is made. Titers above >1,280 is always considered secondary and agreed upon by many others.

### Western blot test

The Western blot (WB) test was first used to identify ribosomal RNA binding proteins in 1979 (Towbin et al., 1979) and is a commonly used technique in biomedical research. There are three major steps in WB test. The proteins are first separated according to their molecular mass, charge, and structure of protein using sodium dodecyl sulfate–polyacrylamide gel electrophoresis (SDS-PAGE) technique. The separated proteins are then transferred and immobilized on to a solid support for the reaction with antibodies, which is a process known as blotting. Finally, the primary and enzyme-conjugated secondary antibodies are used for the detection of proteins. Once detected, the target protein will be visualized as a band on a blotting membrane, X-ray film, or an imaging system (Yang & Mahmood, 2012; Burrell et al., 2017). In comparison to other techniques, WB test has advantages in detecting and semi-quantifying target proteins, allowing the detection of a single target out of a mixture of thousand proteins, obtaining the molecular weight information of the target protein, and can be used as an effective diagnostic tool (Bertoni et al., 2012; Ghosh et al., 2014). The main disadvantage of WB test is that it can only be carried out if the primary antibody against the target protein is available (Gilda et al., 2015).

Hsieh et al. (2021) used the WB test with antigens of ZIKV, WNV, and DENV 1–4 to investigate the antibody responses. Their results showed that the anti-NS1 antibodies to WNV cross-reacted to one or two DENV serotypes, and the anti-DENV NS1 antibodies were unable to differentiate the infecting DENV serotypes in the WB test. Other than that, the anti-E antibodies cross-reacted among these six flaviviruses, while the anti-prM antibodies to DENV serotypes did not cross-react to WNV or ZIKV, which suggested that the anti-DENV prM antibodies can used to distinguish between primary ZIKV infection with previous DENV infection. Moreover, they noted that during the detection of anti-NS1 antibodies, the WB test was more sensitive but less specific as compared to ELISA. This is due to the NS1 protein being present as a hexamer in the solution while NS1 protein is present as a monomer under detergent treatment in WB test, and it is likely that anti-NS1 antibodies can only recognize linear detergent-treated NS1 in ELISA but not recombinant NS1 hexamers, which then reduced the cross-reactivity in ELISA (Ocequera et al., 2007).

### Immunofluorescence test

The indirect immunofluorescence assay (IFA) is a standard virological approach for the detection of antibodies based on their ability to react specifically with viral antigens produced in the infected cells, which are fixed to the wells on a glass microscope slide. Indirect IFA is a two-step assay where the primary unlabeled antibodies in the diluted serum samples will attach to the antigens followed by a fluorophore-labeled secondary anti-human antibody to detect the primary antibody, which then can be visualized using a fluorescence microscope (Sandstrom et al., 1985; Hedenskog et al., 1986; Odell & Cook, 2013). The main advantage of indirect IFA is because the secondary antibody can be used with different primary antibodies, and it is not necessary to conjugate each new antibody individually (Goding, 1996). Furthermore, by combining multiple primary antibodies with specific secondary antibodies (which are tagged with different fluorophore), specific visualization of several antigens can be done simultaneously in one sample (multicolor immunofluorescence). The limitation of the immunofluorescence assay is that the fluorescence signals from the assays are dependent on the concentration and quality of antibody, the appropriate secondary antibodies during the detection, and proper handling of the specimen (Odell & Cook, 2013). The disadvantages of indirect IFA include potential cross-reactivity and the requirement for the primary antibodies that are not generated in the same species or of distinct isotypes when performing multiple-labeling assays (Robinson et al., 2009). An immunofluorescence-based NS1 antigen determination using fluorescein isothiocyanate (FITC) conjugated to IgG antibody was developed and showed a coefficient of determination (R^2^) of 0.92, with a high reproducibility and stability and a low detection limit (LOD) at 15 ng ml^−1^ (Darwish et al., 2018). This optical immunosensor was capable of detecting NS1 analytes in plasma specimens from patients infected with the dengue virus, with low cross-reaction with plasma specimens containing JEV and Zika virus. The major limitation in this assay is that cross-reactivity regarding NS1 specificity was not conducted. The main issues with immunofluorescence assays is with regard to the degradation of fluorochromes, antigens that are not purified adequately, its usage on fixed cells, and also the cost and expertise necessary for this assay.

Currently, indirect IFA is widely used in scientific research rather than for clinical diagnostic purposes. Despite this, IFA was utilized for diagnostic purposes in the early days when ELISA kits were not yet commercially accessible by using patient serum as the primary antibody (Ryu, 2017). IFA have been used for the serodiagnosis of WNV infection (Besselaar et al., 1989), DENV infection (Boopucknavig et al., 1975), and YFV infection (Monath et al., 1981; Niedrig et al., 1999). Research on the evaluation of indirect IFA for the serological diagnosis of DENV in a population with high prevalence of arboviruses was carried out by Arai et al. (2019). They concluded that although the performance of indirect IFA was acceptable, however, for clinical diagnosis of acute infection to detect the IgM antibodies, ELISA alone is sufficient for serological diagnosis. Replacing ELISA with indirect IFA would compromise the sensitivity for IgM and might increase the number of false-negative samples for IgM.

## Conclusion

Despite recent the improvements in *Flavivirus*-specific vaccines, the global burden of *Flavivirus*-associated human diseases is increasing and its area of distribution is expanding. The need for a rapid serological assay that has high sensitivity and specificity is emphasized by the fact that cross-reactive immunity influences the outcome of *Flavivirus* infections. The continued spread of flaviviruses worldwide has resulted in changes in the immune profile, which can change over time, further emphasizing specific diagnosis. Due to the large overlap in clinical disease, accompanied by co-circulation of different flaviviruses, cross-reactivity, and poor access to advance laboratory diagnosis tools for serological confirmation, serological diagnosis of *Flavivirus* infections is a great challenge. The laboratory diagnosis of flaviviruses infection has greatly improved during the last decade. However, serum samples from flaviviruses-infected patients show varying degrees of cross-reactivity to each other when conducting serological diagnosis. Identifying a single antigen with the immunoassay that provides high sensitivity and specificity is one of the difficulties during the diagnosis of *Flavivirus*. Neutralization test is recommended in this review paper for the correct diagnosis of *Flavivirus* of which DENV serotypes showed the least cross-reactivity by using PRNT, and it remains the most common assay used and recommended as the gold standard assay.

## Author contributions

All authors were involved in preparation and writing of the manuscript with CR. Each provided excerpts and comments to all others. All authors contributed to the article and approved the submitted version.

## Funding

This work was supported by the Ministry of Higher Education (MOHE), Fundamental Research Grant Scheme (FRGS) FRGS/1/2021/SKK06/UCSI/01/1

## Conflict of interest

The authors declare that the research was conducted in the absence of any commercial or financial relationships that could be construed as a potential conflict of interest.

## Publisher’s note

All claims expressed in this article are solely those of the authors and do not necessarily represent those of their affiliated organizations, or those of the publisher, the editors and the reviewers. Any product that may be evaluated in this article, or claim that may be made by its manufacturer, is not guaranteed or endorsed by the publisher.
